# Follow-up in breast cancer: does routine clinical examination improve outcome? A systematic review of the literature

**DOI:** 10.1038/sj.bjc.6604065

**Published:** 2007-11-13

**Authors:** D A Montgomery, K Krupa, T G Cooke

**Affiliations:** 1 University Department of Surgery, Level 2, Queen Elizabeth Building, Glasgow Royal Infirmary, Glasgow G31 2ER, UK; 2 Department of Surgery, Royal Alexandra Hospital, Paisley, UK; 3 University Department of Surgery, Glasgow Royal Infirmary, Glasgow, UK

**Keywords:** breast cancer, follow-up, locoregional relapse, detection methods, outcome

## Abstract

Multiple guidelines exist for the follow-up of breast cancer, with no agreement on frequency or duration. The contribution of routine clinical examination for the detection of potentially treatable relapse, and the impact this has on survival, is unknown. In this study, we systematically review the literature to establish the proportion of potentially treatable locoregional relapses and new contralateral breast cancers detected by clinical examination, mammography and patient self-examination. We analyse whether method of detection of relapse influences outcome. The methods used were systematic review of the literature. MEDLINE, EMBASE, CancerLit, Web of Sciences and EBM reviews were the data sources for the systematic review. All studies with information on proportion of relapses detected by clinical examination, mammography and self-examination were included. A total of 30–40% of potentially treatable relapses are detected by patient self-examination. In studies published before 2000, 15% of such relapse is mammographically detected with 46% detected by routine clinical examination. In those published after 2000, 40% are mammographically detected with 15% detected on routine clinical examination. Patients with ipsilateral breast relapse detected clinically appear to do less well than those with relapse detected by self-examination or mammography. Routine clinical surveillance is responsible for detection of fewer potentially treatable relapses in more modern cohorts as experience with mammography increases. There is no evidence to suggest that clinical examination confers a survival advantage compared with other methods of detection. The data in this analysis suggest that a review of the guidelines on follow-up after breast cancer should be undertaken.

The early detection of potentially treatable relapse remains a central purpose of follow-up after breast cancer (National Institute for Clinical Excellence, 2002). Regular clinical examination and mammography are recommended to meet this aim.

Although studies suggest that mammography is useful for detecting metachronous contralateral disease and relapse in the conserved breast at an early and treatable stage (Mellink *et al*, 1991; Grunfeld *et al*, 2002), recent guidelines from the National Institute for Clinical Excellence (NICE) state that the yield of mammography in follow-up is low (National Institute for Clinical Excellence, 2002). The value of clinical examination in detecting locoregional relapse is less certain, yet it is still valued highly by those producing guidelines, who recommend regular clinical examination at least for the first 3–5 years after treatment (American Society of Clinical Oncology, 1999; National Institute for Clinical Excellence, 2002; The Association of Breast Surgery @ BASO and Royal College of Surgeons of England, 2005).

Early detection of locoregional relapse has a beneficial effect on survival (Clark *et al*, 1985; Recht *et al*, 1989; Fowble *et al*, 1990; Kurtz *et al*, 1990; Haffty *et al*, 1991; Dalberg *et al*, 1998). However, it is uncertain whether routine clinical examination contributes to this early detection. Routine clinic visits are stressful for women attending them. Up to 70% of women report feelings of anxiety before such visits (Paradiso *et al*, 1995). While high levels of anxiety are probably attendant on all stages of the process of breast cancer diagnosis and treatment, it is not clear in the case of routine clinic visits whether they are of sufficient value to justify the anxiety they cause.

The aim of this article is to establish, through a systematic analysis of the literature, the relative contributions of clinical examination, patient self-examination and mammography to the detection of potentially treatable relapse (locoregional and new contralateral disease) after breast cancer. The impact on survival of method of detection is also explored.

## MATERIALS AND METHODS

MEDLINE, EMBASE, CancerLit, Web of Sciences and EBM reviews were searched for relevant studies. The original search was conducted in January 2006 and all English language publications between 1966 and January 2006 were considered. Two groups of authors allowed access to further data. Analysis of this further data led to delay in publication of the review and also led to an original report in the literature. To ensure an up to date review and to allow inclusion of the original report in this review without risk of introducing selection bias, the complete search of the literature was repeated in May 2007. The search string used is reproduced as Appendix 1. Both of these initial searches were conducted independently by authors DAM and KK. Titles were studied to assess which abstracts should be obtained. For the remainder of this report, all figures refer to the complete search until May 2007.

All abstracts were read and considered independently by DAM and KK to establish whether the full text article should be retrieved. Sources of disagreement at this stage resulted in the full text article being obtained. References of all full text articles obtained were also searched for further relevant studies.

### Selection criteria

Two separate analyses were conducted; first analysis was a comparison of methods used to detect relapse and a second analysis was the effect of method of detection on survival. Studies were included in a comparison of methods used to detect relapse if:

- The study group comprised women with primary operable invasive breast cancer without metastatic disease out-with the breast and axilla at initial presentation.
- Data pertaining to isolated locoregional relapse only were presented, or such data were presented separately from distant relapse data. Locoregional relapse was defined as relapse within the ipsilateral breast or axilla or new contralateral disease. Supraclavicular lymphadenopathy was considered to be distant disease for the purpose of this analysis.
- Data for each site of relapse analysed are presented separately. There may be differences in the pattern of detection of each site of relapse (ipsilateral breast, contralateral breast or axilla), and this must be fully explored.
- The method of detection (mammography, symptoms or clinical examination) of all types of relapse was included.

The authors of studies which contained some relevant information were written to for extra data, and the study included if the author could provide sufficient data to meet the inclusion criteria above. The studies included in the analysis of methods of detection were included in the further analysis of survival if there was adequate data on survival included in the initial paper, or if the authors were able to supply outcome data after correspondence.

### Assessment of methodological quality of included studies

Methodological quality was assessed independently by two authors (DAM and KK) by means of a pre-defined form. There are no accepted criteria for measuring methodological quality in prognostic studies and so this form was a modified version of the form created by de Bock *et al* (2004), derived from the work by Altman and Lyman (1998) and Laupacis *et al* (1994). The form is presented as Table 1.

When analysing survival, it is of particular concern if patients who are recognised as having relapse are not analysed. In retrospective analyses particularly, this may be because the patient has subsequently died and case notes have been destroyed. Therefore, when assessing the methodological quality in each study, we have included the percentage of patients with recognised relapse not included in the final analysis due to lack of information.

### Data extraction

Two authors, DAM and KK, extracted data from included studies independently. Data collected were year of publication and year of initial operation or referral, population size, age, primary therapy, study design, follow-up schedule including mammographic schedule, number of locoregional relapses and method of detection of locoregional relapse (scheduled *vs* interval clinic and whether detection was by patient, clinician or mammography in the first instance).

Relapse was recorded as clinically detected if it was first detected by a physician in a patient who had not noticed any relevant symptoms. Relapse was recorded as detected by the patient if the patient attended clinic with relevant symptoms, whether the patient waited for the next routine clinic visit or arranged an interval appointment. It was recorded as mammographically detected if an abnormal or suspicious mammogram was recorded before clinical examination revealed any abnormality.

Survival after locoregional relapse was recorded if that information was available.

### Statistical analysis

All data were analysed using SPSS version 11.01 (SPSS Inc., Chicago, IL, USA). For survival calculations, individual data were available for each patient allowing analysis of all individual patients.

## RESULTS

In all, 4061 titles were studied in MEDLINE, 4563 in EMBASE, 8906 in CancerLit and 3144 in Web of sciences. From all EBM reviews including the Cochrane database, three review articles were retrieved. From these titles, 188 abstracts were read and considered independently by DAM and KK. Nine review articles and four letters or editorials were also obtained from the 188 abstracts to examine the references of these articles for further relevant studies. In total, 68 full text articles were considered for inclusion.

From the 68 full text articles considered, 11 studies met the primary inclusion criteria for our analysis of method of detection of locoregional relapse (Mohoney, 1986; Tate *et al*, 1989; Rutgers *et al*, 1991; Hussain *et al*, 1995; Grunfeld *et al*, 1996; Snee, 1996; Lees *et al*, 1997; Jack *et al*, 1998; Churn and Kelly, 2001; Grogan *et al*, 2002; Montgomery *et al*, 2007b). One of these contained information on long-term outcome after local relapse detection (Snee, 1996).

A further 25 studies contained some data appropriate to our meta-analysis and the authors of these were written to. One was able to provide us with further information, including method of detection of local relapse and subsequent outcome for the complete study group and four additional patients, and has therefore been included in both analyses (van der Sangen *et al*, 2006).

From the 12 published studies (Mohoney, 1986; Tate *et al*, 1989; Rutgers *et al*, 1991; Hussain *et al*, 1995; Grunfeld *et al*, 1996; Snee, 1996; Lees *et al*, 1997; Jack *et al*, 1998; Churn and Kelly, 2001; Grogan *et al*, 2002; van der Sangen *et al*, 2006; Montgomery *et al*, 2007b), data were available for 7617 patients with 540 relapses. Seven of these studies (Mohoney, 1986; Rutgers *et al*, 1991; Hussain *et al*, 1995; Jack *et al*, 1998; Grogan *et al*, 2002; van der Sangen *et al*, 2006; Montgomery *et al*, 2007b) analysed patients treated by conservation surgery, two a combination of mastectomy and conservation surgery (Grunfeld *et al*, 1996; Churn and Kelly, 2001) and one mastectomy alone (Snee, 1996). Two studies (Tate *et al*, 1989; Lees *et al*, 1997) did not specify original treatment, but the study by Lees *et al* (1997) includes ipsilateral breast relapse and must therefore have included some patients treated by breast conservation.

Only two studies (Snee, 1996; Montgomery *et al*, 2007b) reported survival related to method of detection of relapse in the original report. One group had published data concerning outcome after locoregional relapse, and were able to supply us with the method of detection for all of these relapses after correspondence (van der Sangen *et al*, 2006). In total, there are 217 locoregional relapses or new contralateral cancers from a cohort of 4625 patients for whom we were able to establish the method of detection of relapse, site of relapse and survival.

Characteristics of all the included studies are presented as Table 2. In particular, Table 2 describes the proportion of relapses detected in the ipsilateral breast, the ipsilateral axilla and the contralateral breast for each included study.

### Quality rating of studies

The median quality score was 6.33 out of 10 with a range from 3 to 9. Five of the 12 studies included new contralateral breast cancers in the analysis. Ten of the 12 included studies analysed all of the locoregional relapses, which they were aware of within their cohort. In the two remaining studies, one failed to analyse 2% of relapses (Rutgers *et al*, 1991) and one failed to analyse 3% of relapses due to inadequate information on method of detection of relapse (Montgomery *et al*, 2007b).

### Method of detection of relapse

The proportion of relapses detected by patient symptoms, mammography and routine clinical examination for each of the studies is presented in Table 3. For Churn and Kelly (2001), data were presented separately for mastectomy and for conservation surgery and so these results are presented separately in the table. Most of the studies were retrospective analyses and in some cases it was not certain how the relapse was detected. These are included in the table as unknown. Relapse detected during further surgery for cosmetic reasons are described as incidental relapses.

Two studies had separate data available to allow analysis of relapse after mastectomy alone (Snee, 1996; Churn and Kelly, 2001). It is not clear what initial surgery was employed in the study by Tate *et al* (1989), but it is likely to have been mastectomy given the date of publication. Clinical examination was an important method of relapse detection in patients after mastectomy, with between 41 and 66% of relapses detected this way. Mammography played no role in the detection of relapse in these studies, as new contralateral disease was not included in any of the analyses. There have been no more recent studies of relapse in patients treated by mastectomy.

Three studies reported on locoregional relapse in a mixed treatment population (Grunfeld *et al*, 1996; Lees *et al*, 1997; Churn and Kelly, 2001). In two studies, it was not possible to separate patients treated by mastectomy from those treated by wide local excision (Grunfeld *et al*, 1996; Lees *et al*, 1997). Discerning a pattern of relapse detection in these studies is difficult as mammography plays a much smaller role in the follow-up of patients treated with mastectomy than in those treated with breast conservation. However, clinical examination detected a smaller proportion of relapses in these studies than in the mastectomy studies, with less than one-third of relapses detected this way.

There were eight studies which looked at the issue of relapse after breast-conserving surgery (Mohoney, 1986; Rutgers *et al*, 1991; Hussain *et al*, 1995; Jack *et al*, 1998; Churn and Kelly, 2001; Grogan *et al*, 2002; van der Sangen *et al*, 2006; Montgomery *et al*, 2007b). In these eight studies, 38% of relapses were detected by the patient, 30% by mammograms and 28% by clinical examination. In 4%, method of detection was unknown.

There was some temporal overlap between these eight studies both with regards to the dates when the included patients had been treated and when relapse was diagnosed. Moreover, the date of relapse of included patients is not always clear in the included studies. The information which does exist is included in Table 2. An attempt was made to assess whether mammography has made a changing contribution to relapse detection over time. Comparison was made of the proportion of relapses detected by each method in the studies of relapse after breast-conserving surgery published prior to 2000, compared with those published after 2000. Date of publication was chosen as a surrogate for date of diagnosis of relapse as it was the only consistent date available for all the published studies (see Table 2). It is likely to underestimate any increase in the importance of mammography as the paper by Churn and Kelly (2001) was published in 2001, yet the analysis included relapse diagnosed only until 1996 and has a pattern of relapse similar to that seen in studies published before 2000. The impact of the study by Grogan *et al* (2002) is limited as so few patients are included. While the proportion of relapses detected by the patient remains fairly constant (39% in studies from those published before 2000, 37% from those published after), the proportions detected by mammography and clinical examination reverse. Before 2000, 15% of relapse was mammographically detected with 46% detected by routine clinical examination. After 2000, 40% is mammographically detected with 15% detected by routine clinical examination.

Three of the studies reported only on ipsilateral breast relapse after wide local excision (Mahoney, 1986; Rutgers *et al*, 1991; van der Sangen *et al*, 2006). In three other studies (Jack *et al*, 1998; Grogan *et al*, 2002; Montgomery *et al*, 2007b), it was possible to extract individual data on method of detection for each area of relapse. Table 4 displays method of detection of ipsilateral breast relapse, Table 5 displays axillary relapses and Table 6 new contralateral breast cancers.

Two patients from Montgomery *et al* (2007b) had simultaneous bilateral breast relapse and so are not included in these tables. Both had their relapses detected by mammography.

As can be seen in Table 4, there is a trend towards increasing proportions of ipsilateral breast relapses being detected by mammography the more recently published the study. In contrast, clinically detected ipsilateral breast relapse becomes less common in more contemporary cohorts.

Very little pattern can be made of axillary relapse, shown in Table 5, as numbers are small. However, the two larger studies in Table 5 are from the same unit and, at least within that unit, fewer axillary relapses are detected by clinical examination in the more recent cohort (Jack *et al*, 1998; Montgomery *et al*, 2007b).

Very few contralateral breast cancers are detected by clinical examination. A total of 66.6% are detected by mammography and 24.4% by the patients themselves.

### Survival

Outcome data were available from three studies within our analysis (Snee, 1996; van der Sangen *et al*, 2006; Montgomery *et al*, 2007b). In one study, primary surgery was mastectomy in all cases (Snee, 1996). In the other two studies (van der Sangen *et al*, 2006; Montgomery *et al*, 2007b), patients were initially treated by wide local excision.

### Mastectomy

Snee reported on five patients who had suffered locoregional relapse after mastectomy (Snee, 1996). Two patients with ipsilateral axillary relapse were diagnosed by clinical examination, one in the breast clinic and one incidentally at another surgical clinic. Three patients had chest wall relapse, two with symptoms and one diagnosed clinically. There was no difference in survival related to method of detection or area of relapse, although three patients died within 2 years of relapse. However, there were very few relapses in this study (Snee, 1996).

### Conservation surgery

van der Sangen *et al* (2006) provided survival data and data on method of detection of relapse for 86 patients who developed ipsilateral breast relapse more than 5 years after wide local excision, method of detection of relapse being unknown in 16 of their cohort. In Montgomery *et al* (2007b), method of relapse detection and survival was known for all 110 patients. Two patients were excluded from this analysis, having had relapse detected incidentally (Montgomery *et al*, 2007b). In total, therefore, method of detection of relapse and long-term outcome is known for 194 patients from these two studies, and is presented below.

### Conservation surgery: ipsilateral breast relapse

**Figure 1:**
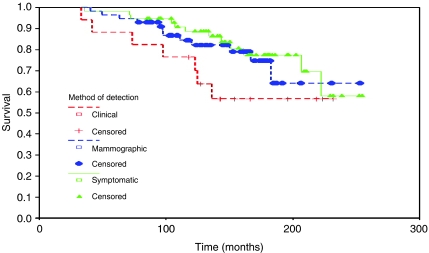
Survival from original operation by method of relapse detection (ipsilateral breast relapses only).

Figure 1 shows survival from time of original operation for all patients with ipsilateral breast relapse only.

**Figure 2:**
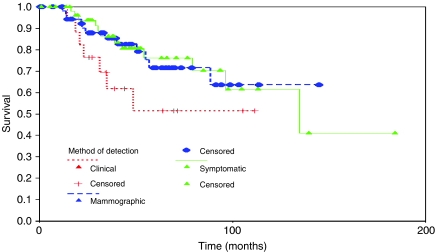
Survival from recurrence by method of relapse detection (ipsilateral breast relapses only).

There is a trend towards lower 10-year survival from original diagnosis in women whose relapse is detected clinically compared with either mammographically detected or detected by the patient, but this difference has disappeared by 15–20 years and overall there is no significant difference in survival between the three groups. This is similar for survival from the time of relapse also (Figure 2).

### Conservation surgery: ipsilateral axillary relapse

There were 25 patients who relapsed in the ipsilateral axilla alone for whom survival data were available. There was no significant difference in overall survival related to method of detection of relapse. There was no difference in time to diagnosis of axillary relapse or in time from relapse to death among the methods of diagnosis either. Numbers were very small in each group, however.

### Conservation surgery: contralateral breast relapse

There were 35 patients with a new contralateral breast cancer in whom survival data were available. Overall 5-year survival from diagnosis of contralateral breast primary was 82.65%.

Twenty-five of these new contralateral cancers were detected mammographically and 5-year survival from diagnosis of new cancer in these patients was 85.8%. There were eight new cancers detected by the patient, with a 5-year survival of 62.5%. There were two women whose new cancer was detected first by clinical examination. Both were alive and well at the time of analysis. There was no significant difference in overall survival or survival from time of diagnosis of new contralateral cancer by method of diagnosis. The number of new cancers diagnosed by means other than mammography was small, however.

## DISCUSSION

Follow-up in breast cancer continues to be an area of importance, both clinically and economically. Current guidelines published by the American Society of Clinical Oncology (ASCO) recommend frequent visits for routine clinical examination and mammography for up to 10 years after treatment (Khatcheressian *et al*, 2006). Clinic attendance for examination was suggested three to four monthly for 3 years, four to six monthly for two further years and then annually for 5 more years. There is particular emphasis on follow-up in the first 3 years after treatment as the rate of relapse has been reported to be particularly high at this time (Hussain *et al*, 1995; Saphner *et al*, 1996).

In contrast to the ASCO guidelines, The National Institute for Clinical Excellence (NICE) in England and Wales recommend that follow-up should be limited to the first 2–3 years after treatment followed by discharge to general practice (National Institute for Clinical Excellence, 2002). NICE estimate the cost savings to be around £3.7 million if follow-up were limited to 5 years, and £9.3 million total savings if follow-up were limited to just 3 years (National Institute for Clinical Excellence, 2002). These cost savings are controversial, however, as they are not balanced by the potential increased costs of late diagnosis of relapse in women who relapse after 3 years.

Both the ASCO and NICE guidelines have in common the fact that emphasis is placed on providing frequent clinical examination in the first 3–5 years after diagnosis. However, this approach is in disagreement with the findings in this review. There certainly is an increase in the hazard rate for relapse in the first 3 years after treatment (Hussain *et al*, 1995; Saphner *et al*, 1996). It is likely that this represents partly a peak in distant relapse and partly a failure on behalf of previous investigators to include new contralateral cancers as a form of relapse. In the analysis by Montgomery *et al* (2007b), there is an initial peak in rate of distant relapse between 2 and 3 years at around 3% of patients per year. This falls to around 2% per year where it remains constant for almost 10 years. In contrast, potentially treatable relapse occurs at a constant rate of 1–1.5% per year for at least 10 years (Montgomery *et al*, 2007b). Since the aims of follow-up as stated by NICE include the detection of potentially treatable locoregional disease only and not distant metastases (National Institute for Clinical Excellence, 2002), there is no justification to discharge at 2–3 years.

However, doubt remains over the value of the ASCO approach of providing regular clinical examination after breast-conservation surgery in the long term. Such examination is responsible for detection of only 13% of the relapses overall in the two most contemporary data sets analysed (van der Sangen *et al*, 2006; Montgomery *et al*, 2007b). Moreover, patients who develop ipsilateral breast relapse that is diagnosed clinically appear to do less well than patients whose relapse is diagnosed by other means. There was significantly reduced survival in patients with clinically diagnosed ipsilateral breast relapse compared with mammographically or self-detected in the study by Montgomery *et al* (2007b). While this was not reflected in our analysis here, this may be because the study by van der Sangen *et al* (2006) did not include relapses diagnosed within the first 5 years after treatment. This group report a very high survival in patients diagnosed more than 5 years after treatment and so method of detection may be less relevant after this time.

This review indicates that in both the conserved breast and the contralateral breast, the contribution of mammography appears not only to be important, but in fact may be of increasing importance. While early studies such as those by Mahoney (1986) reported very few relapse detections using mammography, the proportion of relapses detected this way has increased so that in the studies published since 2000, up to 50% of all treatable breast relapses have been diagnosed first on mammography (Churn and Kelly, 2001; Grogan *et al*, 2002; van der Sangen *et al*, 2006; Montgomery *et al*, 2007b). Further work is needed to assess this in more detail as there was overlap between the studies in terms date of diagnosis of relapse. It would be useful to assess the proportion of relapse diagnosed mammographically each year in a large cohort to confirm the impressions of this analysis.

This change in the impact of mammography is highlighted when comparing two cohorts treated by breast-conserving surgery at the same unit. The number of treatable relapses diagnosed by mammography in Edinburgh has increased from 31% among patients with relapse diagnosed between 1986 and 1998 (Jack *et al*, 1998) to 46% in a more recent cohort diagnosed between 1991 and 2006 (Montgomery *et al*, 2007b). This has arisen from both technical improvements in mammography as well as better quality assurance. As a result, mammography detected 5.37 new cancers per thousand routine mammograms undertaken during follow-up in the most recent Edinburgh cohort (Montgomery *et al*, 2007b), which compares very favourably with the observed rate at incidence screening within the NHS breast screening programme in the UK (NHS Cancer Screening Programmes, 2003). This fully justifies the recommendations in the ASCO guidelines for providing annual mammography, and goes some way to answering the concerns of the Canadian Steering committee who highlight the lack of high quality evidence in the literature on which to base recommendations for mammography (Grunfeld *et al*, 2005).

Interestingly, the proportion of relapses detected by patients, particularly in the treated breast, has remained fairly constant at 30–40% throughout all the studies analysed here. This applies particularly to ipsilateral breast relapse and axillary relapse. It applies less to contralateral breast relapse, where mammography has a much larger impact, and this may reflect high pick up from mammography, less aggressive contralateral disease or simply a lack of patient awareness of the risk of contralateral breast relapse.

There is great disparity between the findings of this analysis and the statements by NICE. NICE base their recommendations for follow-up on the findings of just one retrospective analysis (Donnelly *et al*, 2001), a study which was not included in our analysis due to lack of clarity of the data. The authors of this review report method of detection of relapse in 67 patients who developed metastatic disease and 41 locoregional relapses or new contralateral cancers. Follow-up was for a median of 3 years and 11 months. The authors report that 28 of the locoregional relapses were symptomatic and that 7 were diagnosed clinically. Two were discovered with imaging, but not mammography. The pattern of relapse detection in this cohort is very different from that reported in any of the cohorts we have analysed here.

Much of the data in this review, particularly data relating to survival by method of detection of relapse, have come from unpublished data (van der Sangen *et al*, 2006; Montgomery *et al*, 2007b). There are only 10 studies in the literature which fully present the pattern of relapse after breast cancer with regards to how that relapse is detected. Only one of these (Lees *et al*, 1997) reports on more than 50 patients and only one (Snee, 1996) has any survival data. A recent systematic review of randomised-controlled trials of alternative follow-up methods by ourselves reveals that there are no randomised trials in the literature with sufficient power to inform the guidelines (Montgomery *et al*, 2007a). Previous guidelines have not been based on much evidence.

## SUMMARY

Treatable relapse is not common, affecting only 1–1.5% of women per year (Montgomery *et al*, 2007b). Such relapse occurs at a constant rate after treatment, so the majority of relapses occur more than 3 years after treatment. Patients with later relapses can expect to do particularly well, and so effort should be made to diagnose later relapse at an early stage. If any follow-up for the detection of treatable relapse is to be offered, this cannot stop at 3 years.

The need for clinical follow-up for the detection of relapse is uncertain. The majority of relapses are now detected by patients or mammography. Mammography in fact has a very high yield when conducted annually. There are few relapses detected by clinical examination and, certainly in the case of ipsilateral breast relapse, those which are diagnosed clinically may do less well.

Isolated axillary relapse is very uncommon, and it may be that better patient education could increase the proportion of such relapse detected by the patient. While most women are well schooled in breast self-examination, it may be that the importance of axillary examination is less well appreciated.

Future guidelines should take these facts into account, but should also try to address the additional needs of patients during follow-up for breast cancer. These include both detection of psychosocial problems and side effects of treatment which are central to maintaining patients well being.

## Figures and Tables

**Table 1:** Quality rating of included studies

	**Mahoney (1986)**	**Tate *et al* (1989)**	**Rutgers *et al* (1991)**	**Snee (1996)**	**Hussain *et al* (1995)**	**Grunfeld *et al* (1996)**	**Lees *et al* (1997)**	**Jack *et al* (1998)**	**Churn and Kelly (2001)**	**Grogan *et al* (2002)**	**van der Sangen *et al* (2006)**	**Montgomery *et al* (2007a**, **2007b)**
Is the population under study defined (with inclusion and exclusion criteria)?	Yes	Yes	Yes	Yes	Yes	Yes	No	Yes	Yes	Yes	Yes	Yes
Is the original cohort of patients from which those with relapse were drawn defined?	Yes	Yes	Yes	Yes	Yes	Yes	No	Yes	Yes	Yes	No	Yes
Were all those identified as having relapse analysed?	Yes	Yes	No	Yes	Yes	Yes	Yes	Yes	Yes	Yes	Yes	No
Is loss during follow-up specified?	No	No	No	No	No	Yes	No	Yes	No	Yes	No	No
Are the main prognostic factors defined (at least age of patient and stage of tumour)?	No	No	No	Yes	No	Yes	No	Yes	Yes	Yes	Yes (personal communication)	Yes
Is treatment of first tumour specified (including adjuvant)?	No	No	Yes	Yes	Yes	Yes	No	Yes	Yes	Yes	Yes	Yes
Is mean or median follow-up greater than 5 years?	Not given	No	Not given	Yes	Yes	No	Not given	Yes	Yes	Yes	Yes	Yes
Is the follow-up schedule (including mammographic interval) specified?	Yes	Yes	Yes	No	Yes	Yes	Yes	Yes	Yes	Yes	Yes	Yes
Were methods of diagnosis of relapse prospectively assessed?	Yes	Yes	No	No	No	Yes	No	No	No	No	No	No
Is all relapse, including axillary and new contralateral cancers, included?	Not given	Yes	No	Not given	Not given	Not given	Yes	Yes	No	Yes	No	Yes
Percentage of relapses not analysed due to inadequate information	0	0	2%	0	0	0	0	0	0	0	0	3%
Total score	5	6	4	6	6	8	3	9	7	9	6	7

**Table 2:** Characteristics of studies included in methods of detection meta-analysis

**Study**	**Year**	**Patient group**	**Relapses included**	**Inclusion period**	**Age**	**Primary therapy**	**Study design**	**Follow-up schedule**	**Mammograms**
Mahoney *et al*	1986	273 treated patients	52 locoregional relapses. All ipsilateral breast	All patients treated by lumpectomy between July 1972 and October 1983. All relapses from July 1972 to December 1983 analysed.	Not given	Lumpectomy	Prospective cohort study of the use of thermography	Three monthly for 1 year, four monthly for 1 year, six monthly for 3 years then annual	Biennial
Tate *et al*	1989	510 patients previously treated for early breast cancer attending a follow-up review during the inclusion period.	27 locoregional relapses. Ipsilateral breast (12), Ipsilateral axilla (12) and contralateral breast (3)	6-month period (unspecified) neither original operation dates nor date of relapses specified	At follow-up: mean 65 for interval attenders, 60 for symptomatic routine and 63 for asymptomatic routine	Not specified	Non randomised, non controlled prospective cohort	Two monthly for 1 year, three monthly for 1 year, four monthly for 1 year six monthly for 2 years then annual until 10 years	Not specified
Rutgers *et al*	1991	44 patients with locoregional relapse presenting between 1982 and 1990	44 locoregional relapses All ipsilateral breast	All locoregional relapses diagnosed between 1982 and 1990 from a cohort of all patients treated between 1978 and 1990	mean 47.1 (range: 26–68)	Lumpectomy and axillary dissection	Non randomised, non controlled retrospective cohort	Three monthly for 2 years, six monthly up to 5 years then annual	Annual
Snee	1994	All 33 patients referred to regional centre for adjuvant treatment.	5 locoregional relapses chest wall (3) and axilla (2)	All referrals: jan-feb 1982. Noinformation given on original operation date or period of follow-up scrutinised.	At referral: mean 57 (range=34–78)	Mastectomy	Non randomised, non controlled, prospective cross-sectional	not detailed, mean of two visits each per year.	Not specified
Hussain *et al*	1995	354 treated patients	33 locoregional relapses. Ipsilateral breast (24), Ipsilateral axilla (3) ipsilateral breast and axilla (6). Did not include new contralateral disease	All patients treated between October 1980 and December 1991. Date of analysis not given	Not given	WLE+radiotherapy+at least axillary sample	Non randomised, non controlled retrospective cohort	3 monthly for 2 years, 6 monthly for 3 years then annual until 10 years	6 months then annual
Grunfeld *et al*	1996	296 patients randomised to GP *vs* hospital follow-up.	7 locoregional relapses ipsilateral breast/chest wall (5) and ipsilateral axilla (2)	All patients treated between 1988 and 1992 were randomised to the trial at the end of this period and followed for 18 months from that point	GP follow-up mean 55.6. Hospital follow-up mean 59	153 mastectomy and 138 WLE	prospective randomised comparison of GP v hospital follow-up	three monthly for 1 year and 6 monthly for four in one group, 3, 4 and 6 monthly years 1, 2 and 3 for the other then annual both groups.	Year one then every 1 to 3 years
Lees *et al*	1997	A selected group of 458 treated patients. Selection criteria not given	83 locoregional relapses. All ipsilateral breast	All patients were treated between 1980 and 1985. Follow-up complete until December 1991	Not given	Mastectomy or conservation surgery	Non randomised, non controlled retrospective cohort	three monthly for 2 years then 6 monthly to 5 years then annual	Annual
Jack *et al*	1998	341 treated patients	39 locoregional relapses. Ipsilateral breast (24), ipsilateral axilla (11) and contralateral breast (4)	All patients treated between 1986 and 1990 and followed for 10 years. Date of analysis not given	mean 52.2 (range=24–82)	Wide Local Excision (WLE)+radiotherapy	Non randomised, non controlled retrospective cohort	3-4 monthly for 3 years, then 6 monthly until 10 years	Annual
Churn and Kelly	2001	All 612 patients with early breast cancer referred to regional oncology centre for adjuvant therapy in 1993	34 locoregional relapses. 25 in WLE group and 9 in mastectomy group. Ipsilateral breast, axilla or chest wall (not separated, but did not include new contralateral disease)	All referrals received in 1993 for adjuvant therapy were analysed during 1996	189 patients <50, 423 patients >50	105 mastectomies, 511 conservation, 3 radiotherapy after neo adjuvant chemotherapy. Variable LN dissection	Non randomised, non controlled retrospective cohort	3 to 4 monthly for 2–3 years, 6 monthly to 5 years then annual	Less than annual, according to clinician preference
Grogan *et al*	2002	104 treated patients.	4 salvageable locoregional relapses. Ipsilateral breast (3), Ipsilateral axilla (1) and contralateral breast (0)	Patients treated between January 1988 and June 1991. Follow-up was for 5 years from end of treatment in all patients	Mean 53 (range=28–81)	WLE+radiotherapy	Non randomised, non controlled retrospective cohort	3 monthly for 2 years, 4 monthly for 1 year, 6 monthly thereafter	Annual
van der Sangen *et al*	2006	3280 treated patients. All patients from cohort with locoregional relapse > 5 years after original procedure	102 relapses. All ipsilateral breast	All patients treated between 1982 and 1997. All relapses were between 31 October 1988 and 15 March 2003	Mean 51 (range=32–85)	WLE+radiotherapy	Non randomised, non controlled retrospective cohort	3 monthly for 2 years, 6 monthly for 3 years then annual	Annual (referenced)
Montgomery *et al*	2007	1312 treated patients	110 locoregional relapses ipsilateral breast (45), Ipsilateral Axilla (25), ipsilateral breast and axilla (3), Bilateral breast (1), Bilateral breasts and axilla (1) and contralateral breast (35)	All patients treated between 1991 and 1998. follow-up complete until January 2006	54 (range=24–83)	WLE and either sample or clearance of axilla	Non randomised, non-controlled retrospective cohort	Three to four monthly for 3 years, six monthly to 5 years then annual. Annual for all patients from 2000 onwards	Annual

**Table 3:** Method of detection of all locoregional relapses for all studies

	**Initial surgery**	**Number of patients in study**	**Total number of patients with relapse**	**Patient detected relapses**	**Mammographically detected relapses**	**Clinical examination detected relapses**	**Unknown or incidental relapse detection**
Tate *et al* (1989)	Not given	510	27	16 (59%)	0	11 (41%)	0
Churn and Kelly, 2001	Mastectomy	105	9	1 (12%)	0	6 (66%)	2 (22%)
Snee 1994	Mastectomy	33	5	2 (40%)	n/a	3 (60%)	0
Lees *et al* (1997)	Mastectomy and conservation	438	83	46 (55%)	15 (18%)	22 (27%)	0
Grunfeld *et al* (1996)	Mastectomy and conservation	296	7	2 (28.66%)	2 (28.66%)	2 (28.66%)	1 (14%)
Mahoney (1986)	Conservation	273	52	20 (38%)	1 (2%)	31 (60%)	0
Rutgers *et al* (1991)	Conservation	44	44	26 (59%)	8 (18%)	10 (23%)	0
Hussain *et al* (1995)	Conservation	354	33	4 (12%)	5 (15%)	24 (73%)	0
Jack *et al* (1998)	Conservation	341	39	15 (38%)	12 (31%)	12 (31%)	0
Churn and Kelly, 2001	Conservation	511	25	9 (36%)	7 (28%)	8 (32%)	1
Grogan *et al* (2002)	Conservation	104	4	2 (50%)	2 (50%)	0	0
van der Sangen *et al* (2006)	Conservation	3280	102	41 (41%)	32 (32%)	13 (13%)	16 (16%)
Montgomery *et al* (2007)	Conservation	1312	110	37 (33.5%)	56 (51%)	15 (13.5%)	2 (2%)
Total		7601	540	221 (41%)	135 (25%)	162 (30%)	22 (4%)

**Table 4:** Method of detection of all ipsilateral breast relapses in conservation surgery studies

	**Number of patients in study**	**Total number of patients with relapse**	**Patient detected**	**Mammographic**	**Clinical examination**	**Unknown or incidental**
Mahoney, 1986	273	52	20 (38%)	1 (2%)	31 (60%)	0
Rutgers *et al* (1991)	44	44	26 (59%)	8 (18%)	10 (23%)	0
Jack *et al* (1998)	341	24	9 (37.5%)	9 (37.5%)	6 (25%)	0
Grogan *et al* (2002)	104	3	1 (33%)	2 (66%)	0	0
van der Sangen *et al* (2006)	3280	102	41 (41%)	32 (32%)	13 (13%)	16 (16%)
Montgomery *et al* (2007)	1312	48	17 (36%)	25 (52%)	4 (8%)	2 (4%)

**Table 5:** Method of detection of axillary relapse

	**Patient detected**	**Mammographic**	**Clinical examination**	**Total**
Jack *et al* (1998)	4 (36%)	1 (9%)	6 (55%)	11
Grogan *et al* (2002)	1	0	0	1
Montgomery *et al* (2007)	12 (48%)	4 (16%)	9 (36%)	25
Total	17 (46%)	5 (13.5%)	15 (40.5%)	37

**Table 6:** Method of detection of new contralateral primaries

	**Patient detected**	**Mammographic**	**Clinical examination**	**Total**
Jack *et al* (1998)	2	2	0	4
Grogan *et al* (2002)	0	0	0	0
Montgomery *et al* (2007)	8	25	2	35
Total	10 (26%)	27 (69%)	2 (5%)	39
